# Distinct Expression Patterns of Apoptosis and Autophagy-Associated Proteins and Genes during Postnatal Development of Spiral Ganglion Neurons in Rat

**DOI:** 10.1155/2020/9387560

**Published:** 2020-10-17

**Authors:** Shule Hou, Penghui Chen, Jiarui Chen, Junmin Chen, Lianhua Sun, Jianyong Chen, Baihui He, Yue Li, Huan Qin, Yuren Hong, Shuna Li, Jingchun He, Dekun Gao, Fabio Mammano, Jun Yang

**Affiliations:** ^1^Department of Otorhinolaryngology-Head & Neck Surgery, Xinhua Hospital, Shanghai Jiaotong University School of Medicine, Shanghai, China; ^2^Shanghai Jiaotong University School of Medicine Ear Institute, Shanghai, China; ^3^Shanghai Key Laboratory of Translational Medicine on Ear and Nose diseases, Shanghai, China; ^4^Department of Otorhinolaryngology-Head & Neck Surgery, Shanghai Children's Hospital, Shanghai Jiaotong University, Shanghai, China; ^5^Laboratory of Electron Microscope Center, Shanghai Medical College, Fudan University, Shanghai, China; ^6^Department of Physics and Astronomy “G. Galilei”, University of Padua, Padova, Italy; ^7^Department of Biomedical Sciences, Institute of Cell Biology and Neurobiology, Italian National Research Council, Monterotondo, Italy

## Abstract

Autophagy and apoptosis have a complex interplay in the early embryo development. The development of spiral ganglion neurons (SGNs) in addition to Corti's organ in the mammalian cochlea remains crucial in the first two-week postnatal period. To investigate the roles of apoptosis and autophagy in the development of SGNs, light microscopy was used to observe the morphological changes of SGNs. The number of SGNs was decreased from P1 to P7 and plateaued from P10 to P14. Immunohistochemistry results revealed positive expression of cleaved-caspase3, bcl-2, microtubule-associated protein light chain 3-II (LC3-II), Beclin1, and sequestosome 1 (SQSTM1/P62) in SGNs. The apoptotic bodies and autophagosomes and autolysosomes were also identified by transmission electron microscopy at P1 and P7. Real-time PCR and western blotting results revealed that the apoptotic activity peaked at P7 and the autophagy activity was gradually upregulated along with the development. Taken together, our results for the first time showed that autophagy and apoptosis in SGNs play distinct roles during specific developmental phases in a time-dependent manner.

## 1. Introduction

The auditory function in neonatal rats remains immature, and the cochlear structure and function gradually attain maturity by the second postnatal week. The dendrites of immature spiral ganglion neurons (SGNs) develop innervations to both outer hair cells (OHCs) and inner hair cells (IHCs) simultaneously by the end of pregnancy [1]. During postnatal days 1-7, the immature SGNs are differentiated into type I SGNs and type II SGNs [2]. In addition, the neurites then go through crucial refinement and retraction steps, establishing a specific connection between type I SGNs and IHCs and between type II SGNs and OHCs [1, 3]. Subsequently, the calyx of Held gradually matures in the cochlear nucleus, thereby establishing the function of cochlear output [4]. These bipolar SGNs develop long processes that connect the hair cells (HCs) in the cochlea with the neurons in the cochlear nucleus in order to process accuracy and speed required for auditory information. The specific and appropriate connection between the periphery and central auditory systems is eventually completed at postnatal days 10–12 [5]. The number of SGNs during this period was shown to be reduced [6]. However, the underlying mechanism of transient degeneration and differentiation of SGNs still remains elusive.

Apoptosis (“self-killing”) and autophagy (“self-eating”) are two self-destruction processes that play an important role in the development of inner ear. Also, they are considered important for the maturation of both HCs and SGNs [7, 8], and both apoptosis and autophagy are critical for the survival of HCs and SGNs under varied destruction conditions. Apoptosis is type I programmed cell death that plays a vital role in the development and differentiation of the cochlea [8]. In 2000, Nikolic et al. have used the TdT-mediated dUTP Nick-End Labeling (TUNEL) technique and the results revealed that apoptosis substantially occurs in the cochlear ganglion between E16 and P1 but occasionally occurs between P1 and P14 [9]. However, Echteler and Nofsinger have reported about 27% reduction in the number of SGNs from P3 to P7 [6]. On the other hand, previous reports have shown that autophagy involves a complex connection with apoptosis in the inner ear HCs and SGNs. In the majority of cases, autophagy inhibits cell apoptosis by strengthening cell antistress capacity. Strikingly, autophagy carries out cell death through other pathways and is known as type II or autophagic cell death [10]. Some common and similar stimulus molecules can induce either autophagy or apoptosis or both [11]. However, time-dependent autophagic activities in cochlear SGNs after two weeks of birth have not yet been reported. Therefore, the autophagic and apoptotic characteristic changes during two weeks after birth were evaluated using light and electron microscopy. Furthermore, the autophagic machinery genes such as microtubule-associated protein light chain 3-II (LC3-II), Beclin1, and sequestosome 1 (SQSTM1/P62) and apoptosis-related factors including *bcl-2*, *cleaved-caspase-3*, and *caspase-3* were detected to investigate differential expression patterns of autophagy and apoptotic-associated proteins and genes and their putative correlation during the development of SGNs in rats in the present study.

## 2. Materials and Methods

### 2.1. Animals

Rats (Sprague–Dawley) were purchased from Shanghai SIPPR-BK Laboratory Animals Co. Ltd. All animal operations were in accordance with the guidelines approved by the experimental animal care institution of Xinhua Hospital, Medical College of Shanghai Jiaotong University. In the current experiment, the first postnatal day (P1) was the birthday. P1, P3, P5, P7, P10, and P14 are considered the postnatal time points after the birthday.

### 2.2. Hematoxylin-Eosin (HE) Staining

Cochleas of rats (*n* = 3) (P1-P14) were removed as described previously [12]. The cochleas were 4% PFA fixed, 10% EDTA decalcified, dehydrated by a graded ethanol series, and embedded in paraffin. Then, 3 *μ*m sections were cut and deparaffinized, followed by staining with 75% alum hematoxylin and 0.15% eosin and rehydration with xylene and graded ethanol series.

### 2.3. Transmission Electron Microscope (TEM)

After anesthesia, rats at P1 and P7 underwent cardiac perfusion with ice-cold 2.5% glutaraldehyde (no. G5882, Sigma, USA). The cochleas were dissected and fixed with 2.5% glutaraldehyde for 24 h and decalculated in 10% EDTA. Subsequently, the cochleas were fixed with 1% osmic acid for 2 h at room temperature, rinsed with 0.1 M PBS for 3 times, and dehydrated with ethanol and acetone. The cochleas were immersed in Epon 812. Ultrathin sections (60-70 nm) of cochleas were prepared. Then, the sections were stained with alkaline lead citrate and uranyl acetate and observed under a Philips CM-120 transmission electron microscope (Amsterdam, Philips, The Netherlands).

### 2.4. Immunohistochemical Staining

The paraffin-embedded tissue sections of P7 rat cochlea were blocked in a 1x PBS buffer containing 5% BSA (Sangon Biotech, C500626, China) and 0.3% Triton X-100 (Sigma, no. 30-5140, USA) at room temperature for 1 h, then incubated with the following primary antibodies at 4°C overnight: anti-MAP LC3II mouse monoclonal antibody (1 : 300; Santa Cruz, sc-271625, USA), anti-SQSTM1/P62 recombinant rabbit monoclonal antibody (1 : 200; Abcam, ab109012, USA), anti-Beclin1 rabbit polyclonal antibody (1 : 200; Abcam, ab62557, USA), anti-Bcl2 mouse monoclonal antibody (1 : 200; Servicebio, GB12318, China), anti-cleaved caspase3 rabbit polyclonal antibody (1 : 500; Servicebio, GB11009, China), and anti-caspase3 rabbit polyclonal antibody (1 : 500; Servicebio, GB11009-1, China). After three washes with 0.01 M PBS for 10 mins, the sample sections were incubated with the HRP-conjugated goat anti-rabbit/mouse IgG for IHC (ready to use) (1 : 300; Sangon Biotech, D110073, China) at room temperature for 40 min. After being incubated for 5 mins using a DAB Substrate kit (Sangon Biotech, E670033, China), the specimens were observed under a microscope (Olympus BX43, Tokyo, Japan) and images processed using Adobe Photoshop software.

### 2.5. Quantitative Real-Time PCR

The total RNA of SGNs was extracted by Trizol chloroform isopropanol method, and then, the concentration was determined. cDNA was obtained by using TaqMan® Reverse Transcription Reagents (no. N8080234, ThermoFisher Scientific), and the reaction was carried out in triplicate using SYBR™ Green PCR Master Mix (no. 4344463, ThermoFisher Scientific). *β*-Actin was used as the endogenous reference, and autophagy-related genes *LC3-II*, *P62*, and *Beclin1* and apoptosis-related genes *Bcl2* and *Caspase3* were used as target genes. The primers used for amplification are provided in a list in Table 1. The coefficient of variation about target genes and endogenous reference gene was calculated. The relative expression level of mRNAs was calculated by the 2^-△△CT^ method.

### 2.6. Western Blotting Analysis

Samples were extracted from the isolated P1-P14 cochlear SGNs. The sample proteins were resolved by SDS-PAGE on 12% polyacrylamide gels and transferred to polyvinylidene fluoride (PVDF) membranes (Millipore, USA). The membranes were blocked (Beyotime, China) under ambient temperature for 1 h and probed with the following primary antibodies at 4°C overnight: anti-MAP LC3II mouse monoclonal antibody (1 : 1000; Santa Cruz, sc-271625), anti-SQSTM1/P62 recombinant rabbit monoclonal antibody (1 : 1000; Abcam, ab109012, USA), anti-Beclin1 rabbit polyclonal antibody (1 : 1000; Abcam, ab62557, USA), anti-caspase3 rabbit polyclonal antibody (1 : 1000; Cell Signaling Technology, no. 9662, USA), anti-cleave-caspase3 rabbit monoclonal antibody (Cell Signaling Technology, no. 9664, 1 : 1000, USA), anti-Bcl2 rabbit monoclonal antibody (1 : 2000; Wanleibio, WL01556, China), and GAPDH (1 : 2000; Proteintech, 60004-1-lg, USA). After three washes, the membranes were incubated with a secondary antibody, HRP-labeled goat anti-rabbit IgG or anti-mouse IgG (1 : 1000; Beyotime, A0208/A0216, China), at 37°C for 1 h. Subsequently, the chemiluminescence reagent (Millipore, *A* : *B* = 1 : 1, USA) was utilized to develop the immunoreactive bands, and the images were analyzed using the Bio-Rad ChemiDoc XRS+ (Bio-Rad Company, USA). At least three independent experiments were carried out.

### 2.7. Statistical Analysis

Data are expressed as mean ± SEM, and all experiments were repeated at least three times. Statistical analyses were conducted using GraphPad prism 6 software. Student's *t*-test was used to judge the significant effects between different groups. *P* < 0.05 were considered significantly. Image J software was used to measure the cross-sectional area and to count SGNs to calculate the density of SGNs (the number of SGNs in the cochlea divided by the cross-sectional area).

## 3. Result

### 3.1. Morphological and Quantitative Changes in SGNs during Postnatal Development

**Figure 1:**
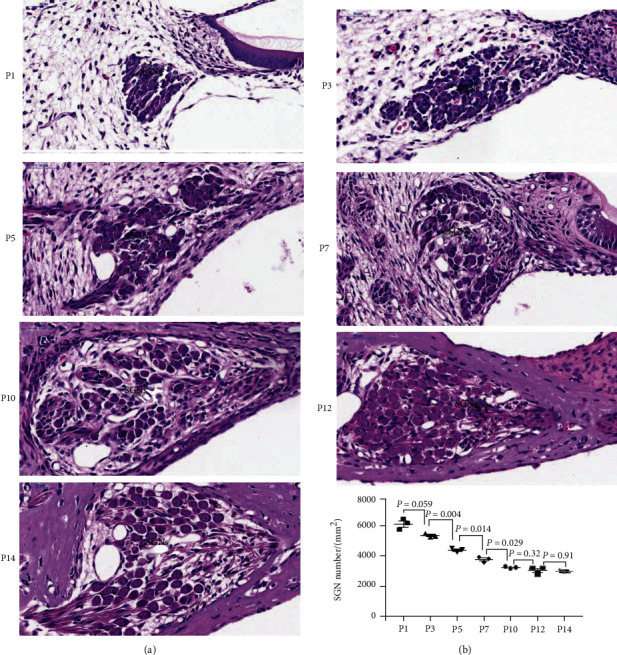
HE staining of SGNs during postnatal weeks from P1 to P14. The SGNs are obtained from the middle turn of the rat cochlea. The immature neurons and glial cells were basophilic and are indicated by blue staining from P1 to P5. The Schwann cells at P12 and P14 were adhered and surrounded by neurons. In the first postnatal week, the density of SGNs was decreased by about 33% and was stabilized in the second week. Scale bars = 50 *μ*m. The number/mm^2^ of SGNs was counted and is shown at the lower part of (b).

The HE staining of SGNs in rats at P1-P14 showed morphological transition at the middle turn (Figure 1). The size of the individual SGN was shown to be significantly increased, and the ratio of the nucleus to the cytoplasm gradually declined from P1 to P14 (Figure 1). The growth rate in the first postnatal week was more than that recorded in the second postnatal week. The density of SGNs was decreased by about 33% during the first week and then plateaued from P10 to P14. The ossification of the bone wall around SGNs started at P7. However, before P7, a distinct boundary between immature SGNs and undifferentiated mesenchymal cells was shown to be absent. The Schwann cells were gradually matured, encompassing the SGN cell body as well as the neurites upon development.

### 3.2. Apoptosis and Autophagy in SGNs under TEM

**Figure 2:**
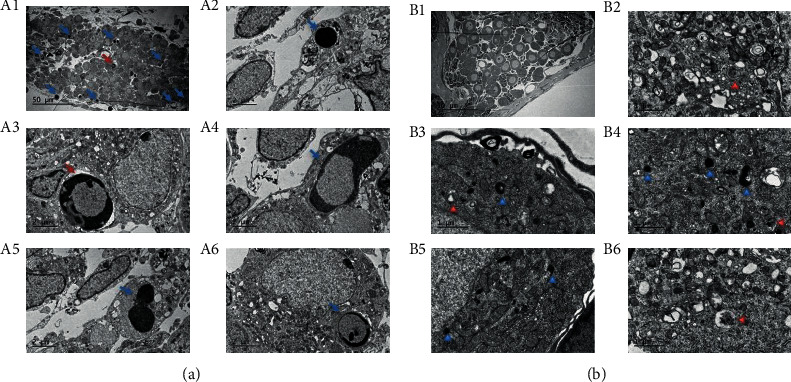
TEM observation of apoptosis and autophagy in SGNs during postnatal development. (A1) The overview of SGNs at P1 showing many apoptotic bodies (blue and red arrow). Scale bars = 50 *μ*m. The morphological differences among various apoptotic cells and nuclear chromatin condensation: (A4) some condensed chromatin moves under the nuclear membrane or (A3) assumes a crescent shape; (A2) some condensed chromatin occupies the whole nuclear area. Scale bars = 2 *μ*m. (A5) The arrow indicates the cell body of secondary necrosis. Scale bars = 2 *μ*m. (B1) The overview of SGNs at P7, scale bars = 50 *μ*m. (B2, B3, B4, and B6) Red arrows indicate autophagosomes, which are presented as double membrane structures and contain a cytoplasm or organelles. (B3–B5) Blue arrows represent autolysosomes. Scale bars = 1 *μ*m.

At P1, several apoptotic bodies were shown as cytoplasmic membrane blebbing, chromatin condensation along the nuclear envelope, chromatin margination, and cell shrinkage (Figure 2(a)), while the nuclear membrane, plasma membrane, and organelles were well preserved. Consecutively, the glial cells and SGNs could not be distinguished (Figure 2(a), A1). However, the apoptotic body is rarely identified at other time points. At P7, the ratio of the nucleus to the cytoplasm in the neurons became evident (Figure 2(b), B1). Also, several autophagic vacuoles and autolysosomes were identified in SGNs at P7 (Figure 2(b), B2–B6).

### 3.3. Immunohistochemical Staining for Apoptosis and Autophagy

**Figure 3:**
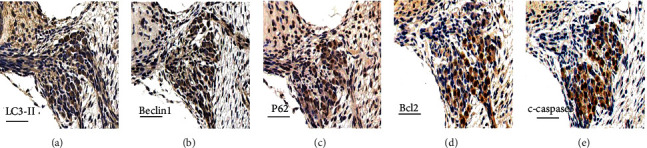
Immunohistochemical staining for autophagy and apoptosis-associated genes at P7. (a), (b) (c), (d), and (e) represent immunohistochemical staining for LC3-II, Beclin1, P62, Bcl-2, and cleaved-caspase3, respectively. The images were obtained from the middle turn of rat cochlea. In the area of SGNs, a positive brown staining was observed in the cytoplasm or nucleus. Scale bars = 50 *μ*m.

Immunohistochemical staining results of LC3-II, P62, cleaved-caspase3, and Bcl-2 showed positive expression of autophagy- and apoptosis-associated proteins in SGNs (Figure 3) at P7.

### 3.4. Real-Time PCR Quantitative Analysis of Apoptosis-Associated Genes and Autophagy Machinery Genes in SGNs during the Postnatal Development

**Figure 4:**
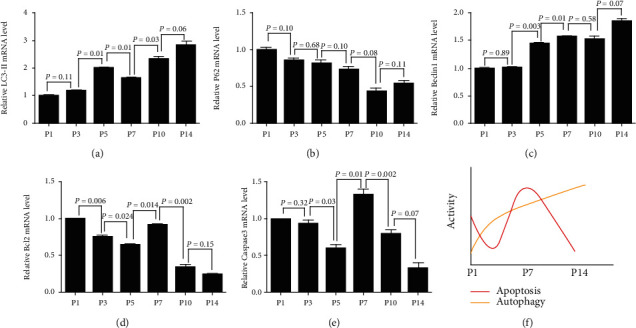
Real-time PCR quantitative analysis of autophagy machinery genes and apoptosis-associated genes in SGNs during the postnatal development. (a–c) The transcription expression of *LC3-II*, *P62*, and *Beclin1* showed that the autophagy activity was upregulated with the development of SGNs. (d, e) The transcription expression of *Bcl2* and *Caspase3* showed maximal apoptotic activity at P7. (f) The overall expression trend of apoptosis and autophagy. Data from triplicate samples were normalized to those at P1. Significant differences were evaluated using repeated measures ANOVA.

The results of real-time PCR quantitative analysis revealed dynamic changes in autophagy machinery genes and apoptosis-associated genes at a transcription level. The expression of *LC3-II* demonstrated an upward trend from P1 to P14, except that it was higher at P5 than at P7 (Figure 4(a)). The expression of *P62* showed gradual declination during these postnatal two weeks (Figure 4(b)), while the expression of *Beclin1* showed an increasing trend (Figure 4(c)). *Bcl2* and *Caspase3* showed similar expression trend during postnatal development. *Bcl2* exhibited maximal expression at P1, which was decreased by 76.3% at P3. The expression of *Bcl2* at P7 was increased by about 1.42-fold when compared to that at P5, followed by a sharp declination by 37.3% at P10 (Figure 4(d)). The expression of *Caspase3* was shown to be decreased at P5, which showed a peak at P7, showing a 2.2-fold decrease when compared to that at P5, and then declined from P7 to P14 (Figure 4(e)). During the postnatal two weeks, the overall expression trend of apoptosis was initially increased and then declined, and autophagy was gradually increased in rat cochlea SGNs (Figure 4(f)).

### 3.5. Western Blot Analysis of Apoptosis-Associated Proteins and Autophagy Machinery Proteins in SGN during the Postnatal Development

**Figure 5:**
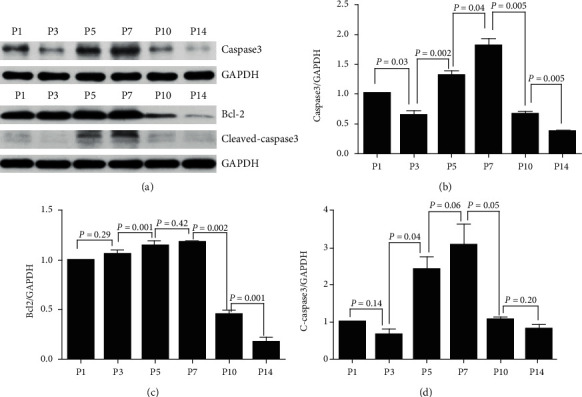
Western blot quantitative analysis for dynamic changes in apoptosis-associated proteins in SGN. (a) The protein expression levels of Bcl2, caspase3, cleaved-caspase3, and GAPDH were measured by western blotting at different developmental time points. (b), (c), and (d) show the half quantitative analysis of Bcl2, caspase3, and cleaved-caspase3 expressions, respectively; and it was normalized to that of GAPDH expression levels. Significant differences were evaluated using repeated measures ANOVA.

**Figure 6:**
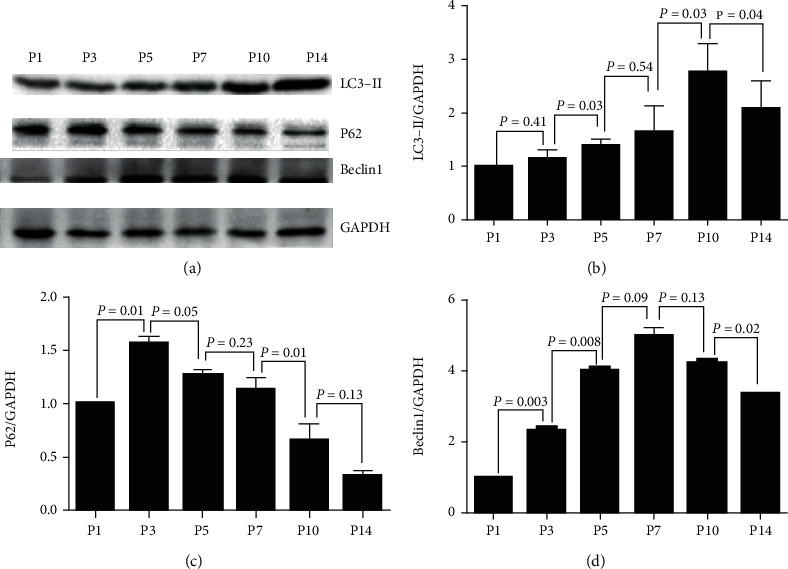
Western blot quantitative analysis for the dynamic change in autophagy machinery proteins. (a) The protein expression levels of LC3-II, P62, Beclin1, and GAPDH were measured by western blotting at different developmental time points. (b), (c), and (d) show the half quantitative analysis of LC3-II, P62, and Beclin1 expressions, respectively, and it was normalized to that of the GAPDH. Significant differences were evaluated using repeated measures ANOVA.

Western blot analysis showed dynamic alteration in apoptosis-associated proteins and autophagy machinery proteins at a protein level. The expression of Bcl-2 was maximized at P7 and sharply declined at P10 and P14 (Figure 5(c)). Caspase3 demonstrated a relatively low expression at P1 and declined further at P3, followed by an increase at P5 and P7 and a sharp decrease at P10 and P14 (Figure 5(b)). Caspase3 and cleaved-caspase3 showed a strong expression at P5 and P7 (Figure 5(c)). The apoptosis-associated proteins expressed maximal apoptotic activity at P7. On the other hand, the expression of LC3-II showed an upward trend from P1 to P10 and declined at P14 (Figure 6(b)). P62 demonstrated low expression at P1, elevated by about 1.5-fold at P3, and gradually decreased from P3 to P14 (Figure 6(c)). The maximal expression of Beclin1 was observed at P7 (Figure 6(d)), which was much earlier than that observed for LC3-II.

## 4. Discussion

HCs in the cochlea play a critical role in the conversion of mechanical sound waves into neural signals, and SGNs transmit these signals to the auditory cortex for hearing. Therefore, HCs and SGNs are considered critical for auditory function. In the mammal's inner ear, HCs and SGNs are vulnerable to multiple damages, but the regenerative ability of HCs and SGNs is limited in mammals, and so most of the damaged HCs and SGNs cannot spontaneously regenerate. Thus, hearing loss in most of the patients is induced by gene mutations, noise, different ototoxic drugs, inflammation, or aging, causing malfunctioning of HCs or SGNs [13]. In the inner ear, apoptosis is genetically aroused in response to various damages, including noise, different ototoxic drugs, inflammation, or aging in HCs and SGNs [13]. The expression of Bcl2, caspase3, and cleaved-caspase3 represents the level of apoptotic activity. Our results showed that the activity of apoptosis peaked at P7 and declined sharply at P10 and P14. Therefore, P7 might be considered a crucial developmental time point. Firstly, Schwartz et al. have found a 27% reduction in the number of SGNs between P3 and P7 [14], which was consistent with that of the apoptotic bodies detected by TEM. Secondly, it has been reported that the neurites underneath the HCs completed refinement and retraction by P8 [3]. Finally, the SGNs begin to differentiate into type I and type II SGNs after P7 [2]. This quantitative alteration of caspase3 suggests that the reduced number of cells between P3 and P7 might be generated by apoptosis due to lack of trophic factors and stimulation from sensory HCs [15]. Although it remains difficult to distinguish whether type I or type II SGNs undergo apoptotic cell death based on the present results, the immature type II SGNs are shown to be in maximum number as they loose afferent innervation from OHCs between P3 and P7 [16].

Autophagy is a critical mechanism of cell death and involves differentiation and development [7, 17]. It is characterized by double-membrane vacuole structures known as autophagosomes. de Iriarte Rodriguez et al. have demonstrated a twofold *Beclin1* and *Atg9a* mRNA expression at P30 when compared to that at P0 [18], suggesting the upregulation of autophagic activity with growth during postnatal development. However, the expression trend of these genes during P0–P14, which is a key phase of development, remains unclear. In this study, the real-time PCR results showed that the expression of *LC3-II* and *Beclin1* was upregulated, while that of *P62* was downregulated in cochlear SGNs from P1 to P14. Western blotting analysis results revealed that the maximal expression of LC3-II and Beclin1 proteins in cochlear SGNs was at P10 and P7, respectively.

A previous study conducted in chicken showed that the inhibition of *LC3-II* resulted in the accumulation of apoptotic cells in otic vesicles [19]. These results supported that autophagy provided energy for removing the damaged organelles or apoptotic cells and is regarded essential for the migration of neural precursors. Additionally, Kuma et al. have found decreased levels of amino acids in the plasma and adipose tissues of *Atg5*-, *Atg7*-, *Atg9*-, and *Atg16*-mutant mice, and these mice died shortly after birth, suggesting that autophagy serves as an energy source during the perinatal period [15]. Generally, the upregulated autophagic activity during the first postnatal week induces energy for undergoing apoptosis, and this peaks at P7. Moreover, with the increasing cell size and the ratio of the cytoplasm to the nucleus along with the development, a large number of proteins and organelles are required for generating high-level autophagy. In terms of differentiation, active autophagy facilitates the turnover of specific receptors and factors in order to promote different cell fates [7]. In addition, the maturely differentiated neurons might face various external injuries, and therefore, autophagy plays a homeostatic role in refreshing intracellular components and resistance to external stress [16].

More importantly, the correlation between apoptosis and autophagy might be more complicated than speculated [20]. Autophagy and apoptosis are triggered by common upstream signals that occasionally combine with the processes [21]. In other patients, the cell switches between the two responses in a mutually exclusive manner [22]. Thus, the purpose of distinct expression patterns of autophagy- and apoptosis-associated proteins and genes during the two postnatal weeks is to satisfy the requirement for the development of SGNs. However, the molecular machinery underlying the change in apoptosis and autophagy still remains unclear.

In summary, the present morphological experiment revealed reduction in the number of SGNs and increment in cell size during postnatal development. For the first time, the time-dependent dynamic alteration of apoptosis and autophagy from P1 to P14 in rat cochlear SGNs was examined. The results of apoptosis reached the peak at P7, and autophagy reached the peak at P10 or in the later stages. This suggested that both apoptosis and autophagy play distinct roles during different developmental time points, although the significance of inconsistencies at these time points that show peak in the expressions remained unclear. Therefore, additional studies are warranted to clarify the correlation between apoptosis and autophagy as well as the underlying mechanisms during cochlear development.

## Figures and Tables

**Table 1:** Primers for real-time-PCR.

Gene	Forward (5′-3′)	Reverse (5′-3′)
*LC3 II*	ATCAACATTCTGACGGAGCGG	ATCTGCCTGCTTGTCCTGGTT
*P62*	TGTCTTGGGGAAGGGTTCGAT	GCATAAGCTTCACATGGGGGT
*Beclin1*	CCTCTGAAACTGGACACGAGC	GCTGGGGGGATGAATCTTCGA
*Bcl2*	TCTTTGAGTTCGGTGGGGTCA	AGTTCCACAAAGGCATCCCAG
*Caspase3*	GAAAGCCGAAACTCTTCATCAT	ATGCCATATCATCGTCAGTTCC
*β-Actin*	TGCTATGTTGCCCTAGACTTCG	GTTGGCATAGAGGTCTTTACGG

## Data Availability

All data used during the study are available from the corresponding author by request.
